# Primary pulmonary malignant melanoma: a clinicopathologic study of two cases

**DOI:** 10.1186/1746-1596-7-123

**Published:** 2012-09-19

**Authors:** Li Gong, Xiao-Yan Liu, Wen-Dong Zhang, Shao-Jun Zhu, Li Yao, Xiu-Juan Han, Miao Lan, Yan-Hong Li, Wei Zhang

**Affiliations:** 1 The Helmholtz Sino-German Laboratory for Cancer Research, Department of Pathology, Tangdu Hospital, The Fourth Military Medical University, Xi’an, 710038, P.R China; 2 Department of Gynaecology and Obstetrics, Tangdu Hospital, the Fourth Military Medical University, Xi’an, 710038, P.R China; 3 Department of Pathology, Tangdu Hospital, The Fourth Military Medical University, Xi’an, 710038, P.R China

**Keywords:** Primary pulmonary melanoma, Surgical resection, Chemotherapy, Metastasis, Treatment

## Abstract

**Abstract:**

Malignant melanoma involving the respiratory tract is nearly always metastatic in origin, and primary tumors are very rare. To our knowledge, about 30 cases have been reported in the English literature, one of which involved multiple brain metastases. Here, we report two cases of primary pulmonary malignant melanoma. The first case, which occurred in a 52-year-old Chinese female patient who died 4 months after the initial diagnosis, involved rapid intrapulmonary and intracranial metastases. The second patient, a 65-year-old female, underwent surgical excision, and clinical examination, histopathological characteristics, and immunohistochemical features supported the diagnosis of pulmonary malignant melanoma. No evidence for recurrence and/or metastasis has been found more than one year after the initial surgery. To establish the diagnosis of primary pulmonary malignant melanoma, any extrapulmonary origin must be excluded by detailed examination. Moreover, the tumor should be removed surgically whether it occurs as a single lesion or multiple lesions.

**Virtual slide:**

The virtual slide(s) for this article can be found here: http://www.diagnosticpathology.diagnomx.eu/vs/1480477335765055.

## Introduction

Malignant melanoma occurs most frequently on the skin, but can also arise in other organs and tissues of the body. However, primary pulmonary malignant melanoma is exceedingly rare [1]. To date, about 30 cases have been reported in the English literature [2]_,_ one of which involved multiple brain metastases [3]. This report presents two cases of primary malignant melanoma of the lung, and one case involves intrapulmonary and intracranial metastasis. The goal of this study was to illustrate the importance of establishing the diagnosis of primary pulmonary malignant melanoma by detailed examination upon diagnosis to exclude any extrapulmonary origin. Moreover, the tumor should be removed surgically whether it occurs as a single lesion or multiple lesions.

## Case presentation

### Case 1

**Figure 1:**
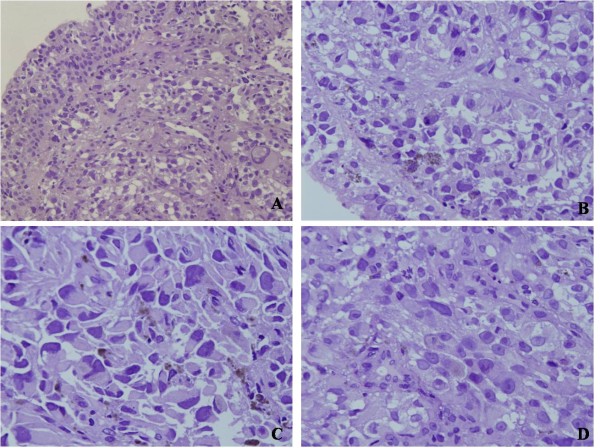
**Histopathological characteristics of pulmonary malignant melanoma: The tumor cells located under bronchial mucosa, and was composed of malignant epithelioid tumor cells with large amounts of acidophilic cytoplasm and prominent nuclei.** Mitotic figures were easily found. Dark brown pigment were observed in some tumor cells (**A,** ×200; **B,C,** and **D,** ×400).

**Figure 2:**
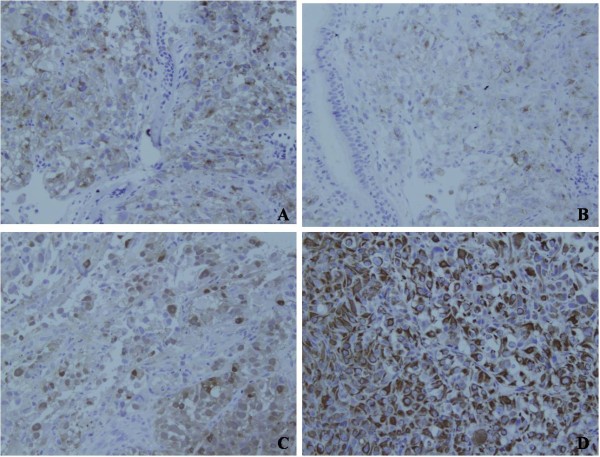
Immunohistochemical features of pulmonary malignant melanoma: The results of immunohistochemical staining demonstrated the tumor cells were positive for pan- Melanoma (Figure 2A, ×200), HMB45 (Figure 2B, ×200), S-100 protein (Figure 2C, ×200), and Vimentin (Figure 2D, ×200).

A 52-year-old Chinese female was admitted to our hospital because of a persistent cough. Previous computed tomography (CT) of the chest revealed multiple ill-defined masses in the upper and lower lobe of the left lung. Clinical examinations and routine laboratory tests were within normal limits. Comprehensive dermatologic and ophthalmologic examinations revealed no evidence of cutaneous or ocular primary melanoma. Bronchoscopic examination showed a bulging lesion in the bronchus of the lower left lobe, and a bronchial mucosal biopsy was performed. Histopathologically, the tumor cells were submucosal, and were composed of malignant epithelioid cells with large amounts of acidophilic cytoplasm and prominent nuclei. Mitotic figures were easily found. Dark brown pigment was observed in some tumor cells (Figure 1). Thus, our initial diagnosis was malignant melanoma. Immunohistochemical staining to confirm this diagnosis demonstrated that the tumor cells were positive for pan melanoma, S-100 protein, Vimentin, and HMB45 (melanoma marker) (Figure 2), and were negative for cytokeratin (CK), epithelial membrane antigen (EMA), chromaffin A (CgA), synaptophysin (Syn), high molecular weight cytokeratin, small cell lung cancer (SCLC) , Desmin, smooth muscle actin (SM-actin), neuron specific enolase (NSE), leukocyte common antigen (LCA), and CD34. Based upon these histological characteristics, immunohistochemical features, and the fact that there was no evidence of malignant melanoma elsewhere, the final diagnosis was primary pulmonary malignant melanoma. The patient underwent regular adjuvant chemotherapy with dacarbazine. Three months after the initial diagnosis, the patient felt intermittent dizziness, and a cranial CT showed a solitary lesion in the occipital lobe. The physician thought that the intracranial lesion might be a metastatic focus, and advised the patient to begin therapy. However, she refused to undergo treatment and left hospital. At the end, she died 4 months after the initial diagnosis.

### Case 2

**Figure 3:**
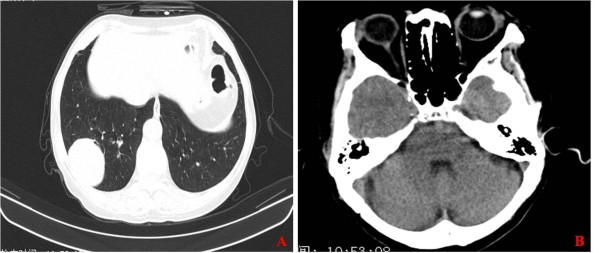
Chest and cranial CT of the second patient: A chest Computed tomography (CT) revealed a spherical mass in the right lung lower lobe (Figure 3A), and cranial CT showed normal (Figure 3B).

**Figure 4:**
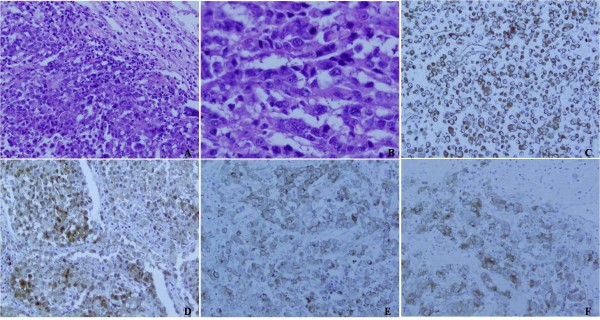
**Histopathological characteristics and immunohistochemical features of the second case of pulmonary malignant melanoma: Histopathologically, the tumor was composed of malignant epitheliod tumor cells with large amounts of acidophilic cytoplasm and prominent nuclei.** Mitotic figures were easily found. Lung tissue could be found in the surrounding of tumor cells (Figure 4**A** ×200and 4**B** ×400). Immunohistochemically, the tumor cells were positive for vimentin (Figure 4**C** ×200), S-100 protein (Figure 4**D** ×200), melanoma-pan (Figure 4**E** ×200), and HMB45 (Figure 4**F** ×200).

A 65-year-old Chinese female was admitted to our hospital due to shortness of breath and weakness of 8 months’ duration. She was initially treated with antibiotics for 1 week at home. However, her condition did not improve, and she was admitted to our hospital for further studies. A chest CT revealed a spherical mass in the lower lobe of the right lung. Her cranial CT was normal (Figure 3). Clinical examinations and routine laboratory tests were within normal limits. The patient demanded and received surgical treatment. Grossly, the mass measured 6×4×4 cm, the cut surface was dark, and the texture was solid. Histological examination revealed the features of malignant melanoma with predominantly epitheliod cells and nuclei (Figure 4). This was further confirmed by immunohistochemistry, which showed that the tumor cells expressed pan melanoma, HMB45, and S-100 protein (Figure 4). As the same as the first patient, various examinations demonstrated that there was no lymph node metastasis or evidence of melanoma elsewhere. The final diagnosis was primary pulmonary malignant melanoma. Thus, the patient began adjuvant chemotherapy with dacarbazine three weeks after operation. To date, she is alive one year and six months after initial diagnosis. Moreover, there is no evidence of recurrence and/or metastasis.

## Discussion

Worldwide, approximately 160,000 new cases of melanoma are diagnosed each year, and about 41,000 melanoma-related deaths occur annually [4]. Malignant melanoma mainly occurs on the skin, but has also been described in other mucosal sites and organs, including the oral cavity, paranasal sinuses, esophagus, larynx, vagina, anorectal region, and liver [5,6]. Primary malignant melanoma of the lung is an extremely rare non-epithelial neoplasm that accounts for only 0.01% of all primary lung tumors [7]. To date, about 30 cases have been reported in the English literature [2]. The mean age at diagnosis is 57 years (range 41–82). Although it appears to be dominant in males, both cases described in this report were female.

The precise histogenesis of pulmonary malignant melanoma remains controversial. Most experts believe that melanocytes migrate concomitantly with reduced growth of the primordial tubular respiratory tract during fetal growth [8]. Others think that these cases are a metastatic form of an antecedent skin lesion that is either unrecognized or has spontaneously regressed [9]. In addition, there are some likely explanations regarding the presence of melanoma in the lung, such as the possibility that melanocytes and melanocytic proliferations are present in the larynx and esophagus, or that the larynx, esophagus, and lungs all share a common embryologic origin, suggesting the possible migration of melanocytes [10].

Pathologically, primary pulmonary malignant melanoma resembles that of the skin or mucosa, and exhibits morphologic variability within the tumor sample. Microscopically, the tumor is composed of epithelioid cells arranged in nests, or spindle cells arranged in fascicles, with or without melanin pigment deposition. Mitotic figures are readily apparent. In both of our cases, the tumor cells were mainly submucosal and showed diffuse infiltration. They were pleomorphic, with round, spindle-shapes, irregular morphologies, and prominent nuclei. Some tumor cells contained melanin deposits. Thus, in similar cases, malignant melanoma should be the first consideration. However, immunohistochemical staining should also be performed to further confirm this diagnosis, and to exclude other melanotic tumors, such as melanotic medullary carcinoma of thyroid [11], and pigmented neuroendocrine carcinoma [12]. In our two cases, immunohistochemical staining demonstrated that the tumor cells expressed HMB45, S-100, pan melanoma and Vimentin, and did not express CK, EMA, CgA, Syn, HCG, HMW-CK, Desmin, SM-actin, TTF-1, and SCLC. Thus, the diagnosis was reliable, although there was no evidence of melanoma from transmission electron microscopic examination.

According to the published literature, approximately 5-10% of patients with metastatic melanoma have a primary melanoma of unknown origin [13,14]. Various reasons, such as occult cutaneous or visceral location, complete regression, or primary origin in lymph nodes due to malignant transformation of ectopic nevus cells, have been suggested [15,16]. Multiple nodules of the lung are generally considered intrapulmonary metastases. There were multiple lesions in our first case, so we had to determine whether the tumor was a primary or secondary lesion. To this end, we performed an extensive examination for the patient, including physical examinations, gastrointestinal endoscopy, colonoscopy, endoscopy of the nasalcavity, and positron emission tomographic scanning of the brain. The results showed no evidence of malignant melanoma elsewhere. In addition, CT did not reveal a solitary lesion in the occipital lobe upon initial admission. Moreover, according to the clinical and pathological criteria proposed by Allen and Drash and others [1,8,17]– 1) no history suggestive of a previous melanoma; 2) no demonstrable melanoma in any other organ at the time of surgery; 3) a solitary tumor in the surgical specimen from the lung; 4) tumor morphology compatible with that of a primary tumor; 5) no evidence at autopsy of a primary melanoma elsewhere; 6) obvious melanoma cells confirmed by immunohistochemical staining for S-100 and HMB-45, and possibly by electron microscopy; 7) evidence of junctional change; 8) “nesting” of cells beneath the bronchial epithelium; 9) invasion of the intact bronchial epithelium by melanoma cells – we concluded that the aforementioned data were indicative of primary lung melanoma with intrapulmonary metastasis. Moreover, we considered that the ninth view described in the above diagnostic criteria was an important characteristic of primary pulmonary melanoma besides the evidence of histopathological and immunohistochemical staining according to the features of our both cases. The tumor cells of primary pulmonary melanoma seemed also to involve in the bronchial epithelium.

The optimal treatment for patients with primary malignant melanoma of the lung remains to be determined. Some studies have demonstrated that the prognosis for surgically-resected patients is better than that for nonsurgically treated patients [2]. Our second case confirms this viewpoint. Of course, various chemotherapeutic agents, including dacarbazine and immunotherapy with interleukin-2 or interferon should be used as well. However, a cranial CT revealed a solitary lesion in the occipital lobe 3 months after the initial diagnosis for the first patient. We concluded that this was a probable metastatic malignant melanoma though a biopsy was not performed. Therefore, we feel that an aggressive surgical approach is warranted.

## Conclusion

In conclusion, the extrapulmonary origin of malignant melanoma must be excluded by detailed examination to establish the diagnosis of primary pulmonary malignant melanoma. The tumor should be removed surgically whether it occurs as a single lesion or multiple lesions.

## Consent

Written informed consent was obtained from the patients for publication of this report and any accompanying images.

## Abbreviations

CgA: Chromaffin A; CK: Cytokeratin; CT: Computed tomography; EMA: Epithelial membrane antigen; HMW-CK: High molecular weight-cytokeratin; SCLC: Small cell lung cancer; LCA: Leukocyte common antigen; NSE: Neuron specific enolase; SM-actin: Smooth muscle actin; Syn: Synaptophysin.

## Competing interests

The authors declare that they have no competing interests.

## Authors’ contributions

GL conceived of the study and drafted the manuscript. LXY, YL, ZSJ, and HXJ participated in its acquisition of data and analysis. ZWD participated in drafting the manuscript. LM carried out the immunohistochemical assays. ZW and LYH participated in its design and coordination and helped to draft the manuscript. All authors read and approved the final manuscript.
